# Prognostic group stratification and nomogram for predicting overall survival in patients who received radiotherapy for abdominal lymph node metastasis from hepatocellular carcinoma: a multi-institutional retrospective study (KROG 15-02)

**DOI:** 10.18632/oncotarget.21775

**Published:** 2017-10-10

**Authors:** Youngkyong Kim, Hee Chul Park, Sang Min Yoon, Tae Hyun Kim, Jieun Lee, Jinhyun Choi, Jeong Il Yu, Jin-Hong Park, Jong Hoon Kim, Joong-Won Park, Jinsil Seong

**Affiliations:** ^1^ Center for Liver Cancer, National Cancer Center, Goyang, Korea; ^2^ Department of Radiation Oncology, Samsung Medical Center, Sungkyunkwan University School of Medicine, Seoul, Korea; ^3^ Department of Radiation Oncology, Asan Medical Center, University of Ulsan College of Medicine, Seoul, Korea; ^4^ Department of Radiation Oncology, Yonsei University College of Medicine, Seoul, Korea

**Keywords:** hepatocellular carcinoma, lymph node metastasis, survival, prognostic factor, nomogram

## Abstract

**Objective:**

To develop a prognostic model for overall survival (OS) in hepatocellular carcinoma (HCC) patients receiving radiotherapy (RT) to metastatic abdominal lymph nodes (LNs).

**Materials and Methods:**

Two hundred twenty-eight patients treated with RT to metastatic abdominal LNs were retrospectively reviewed.

**Results:**

Median OS in all patients was 11.1 months. LN responders had significantly higher median OS than non-responders (14.2 months vs. 7.5 months, *p*<0.05). On multivariate analysis, Child-Pugh classification, status of intrahepatic tumor, presence of distant metastasis, number and location of metastatic LNs, serum level of alpha fetoprotein (AFP), and the LN response to RT were significant prognostic factors for OS (*p* < 0.05 each). Based on the results of multivariate analysis, prognostic group stratification according to the number of pre-treatment risk factors was a significant predictor of OS, and median OS in patients with ≥ 4, 3, 2, 1, and 0 risk factors were 2.9, 5.5, 10.3, 13.6, and 27.8 months, respectively (*p*<0.05). A nomogram was formulated by integrating the different prognostic contribution of each factor, and it showed good accuracy for predicting 2-year OS with a concordance index of 0.72.

**Conclusion:**

Prognostic group stratification and nomogram could be useful prognostic and therapeutic indicators in selecting treatment strategies.

## INTRODUCTION

Abdominal lymph node (LN) is a frequent site of extrahepatic metastasis from hepatocellular carcinoma (HCC), along with lung and bone [1–4], with a reported incidence of 25 to 42% in several autopsy series [2, 4], and it is associated with poor prognosis. With advances in diagnostic imaging technology and improvements of intrahepatic tumor control by effective local treatments, abdominal LN metastasis from HCC may become more frequent in clinical situations. Various treatment modalities, such as surgical resection, transcatheter arterial chemoembolization, radiofrequency ablation, and percutaneous ethanol injection, have not been considered as an option in most cases due to the presence of intra- and extrahepatic tumors and/or poor hepatic function, insufficiency of blood supply, and the closeness of the gastrointestinal organs, respectively [1–19]. Moreover, although sorafenib, an oral multikinase inhibitor, significantly prolongs survival in patients with advanced HCC in two large randomized phase III clinical trials, it has not shown survival benefits in subgroups with extrahepatic metastases [8, 9]. Fortunately, RT for metastatic LNs from HCC has been reported to be a feasible option, with promising response and increased survival [10–14, 16–18], but the benefit of RT to abdominal LN metastasis has remained unclear due to the limitations of retrospective study.

Inherently, the survival of patients with metastatic LNs from HCC is thought to be influenced by patient factors (i.e., hepatic function), and LN or intrahepatic tumor-related factors (i.e., size, number and location of metastatic LNs, status of intrahepatic tumors, presence of distant metastases). Thus, better understanding of the influence of these tumor and patient factors on prognosis is helpful in selecting treatment strategies to treat metastatic abdominal LN from HCC, such as supportive care, surgical resection, RT, sorafenib, and aggressive multimodal approaches. However, to date, the reported prognostic factor for survival varied among studies, the numbers of patients in those studies were relatively small, and the studies were single institutional [10–15, 17–19]. Considering the relative rarity of the clinical situation of RT for abdominal LN metastasis from HCC, it has been impossible to evaluate the efficacy of RT and analyze the effect of tumor and patient factors on prognosis through a large-scale prospective study. Thus, we performed this retrospective multi-institutional study with subjects obtained from four institutions of the Korean Radiation Oncology Group (KROG) to analyze the effects of pretreatment tumor and patient factors on clinical outcomes in HCC patients who underwent RT to treat metastatic abdominal LN(s) and to identify prognostic and therapeutic indicators in these patients for developing the prognostic model to predict survival.

## RESULTS

### Patient characteristics

The patient characteristics are summarized in Table 1. There were 194 men and 34 women, and the median age was 59 years (range, 31 - 81 years). The median interval between the diagnosis of the intrahepatic tumor and diagnosis of LN metastasis was 12.1 months (range 0.0 - 154.3 months) and synchronous LN metastases were observed in 52 patients (22.8%). Of 228 patients, 153 (67.1%) had regional abdominal LN(s), and 106 patients (46.5%) presented with ≥ 3 metastatic LNs. At the time of RT for metastatic abdominal LN(s), 147 patients (64.5%) had controlled intrahepatic tumor, being in remission or with stable disease, without any clinical, laboratory, or radiological findings suggestive of intrahepatic tumor progression at 2 months before RT, and 47 patients (20.6%) had distant metastasis, except for metastatic abdominal LNs, including metastases to the bones (n=17), lungs (n=15), non-abdominal LNs (n=12), peritoneal seeding (n=8), and adrenal glands (n=2). The median total radiation dose was 46 Gy (range, 30 - 60 Gy), with fraction sizes of 1.8 - 9 Gy. Due to the diversity of fraction size, EQD2 was used for analysis, and the median EQD2 was found to be 46.7 Gy_10_ (range, 30 - 80 Gy_10_). Of 228 patients, 168 (73.7%) underwent 3DCRT, 59 (25.9%) underwent IMRT and 1 (0.4%) underwent SBRT. The median EQD2 for the treatment techniques was 44 Gy_10_ (range, 30 - 71 Gy_10_) for 3DCRT, 61 Gy_10_ (range, 32.5 - 80 Gy_10_) for IMRT, and 57 Gy_10_ for SBRT.

**Table 1:** Patient and tumor characteristics (n = 228)

Characteristics		Total, n (%)	LN response, n (%)		*p* value
			Responder	Non-responder	
Gender	Male	194 (85.1)	129 (66.5)	65 (33.5)	0.592^∥^
	Female	34 (14.9)	21 (61.8)	13 (38.2)	
Age (years)	Median (range)	59 (31-81)	58.5 (36-81)	59.5 (31-78)	0.473^¶^
	< 60	118 (51.8)	79 (66.9)	39 (33.1)	0.702^∥^
	≥ 60	110 (48.2)	71 (63.5)	39 (35.5)	
ECOG PS	0-1	217 (95.2)	144 (66.4)	73 (33.6)	0.517^∥^
	2-3	11 (4.8)	6 (54.5)	5 (45.6)	
Etiology of LC	HBV	152 (66.7)	94 (61.8)	58 (38.2)	0.076^∥^
	Others	76 (33.3)	56 (73.7)	20 (26.3)	
Child-Pugh classification	A	196 (86.0)	133(67.9)	63 (32.1)	0.103^∥^
	B	32 (14.0)	17 (53.1)	15 (46.9)	
AFP (ng/mL)	Median (range)	34 (1-349833)	19 (1-129504)	76 (1-349833)	0.145^¶^
	<400	168 (73.7)	114 (67.9)	54 (32.1)	0.271^∥^
	≥400	60 (26.3)	36 (60.0)	24 (40.0)	
Vascular invasion	No	169 (74.1)	119 (70.4)	50 (29.6)	0.013^∥^
	Yes	59 (25.9)	31 (52.5)	28 (47.5)	
Status of intrahepatic tumor	Controlled	147 (64.5)	101 (68.7)	46 (31.3)	0.211^∥^
	Uncontrolled	81 (35.5)	49 (60.5)	32 (39.5)	
Synchronicity of metastatic LN	No	176 (77.2)	120 (68.2)	56 (31.8)	0.161^∥^
	Yes	52 (22.8)	30 (57.7)	22 (42.3)	
Size of metastatic LN (cm)	Median (range)	2.9 (0.5-8.5)	2.8 (0.6-8.5)	3.3 (0.5-8.5)	0.100^¶^
	≤ 3	115 (50.4)	82 (71.3)	33 (28.7)	0.077^∥^
	> 3	113 (49.6)	68 (60.2)	45 (39.8)	
No. of metastatic LN	1	80 (35.1)	59 (73.8)	21 (26.2)	0.124^∥^
	2	42 (18.4)	28 (66.7)	14 (33.3)	
	≥ 3	106 (46.5)	63 (59.4)	43 (40.6)	
Location of metastatic LN	Regional	153 (67.1)	105 (68.6)	48 (31.4)	0.197^∥^
	Non-regional	75 (32.9)	45 (60)	30 (40)	
Distant metastasis	Absent	181 (79.4)	120 (66.3)	61 (33.7)	0.751^∥^
	Present	47 (20.6)	30 (63.8)	17 (36.2)	
Previous treatment	No	4 (1.8)	3 (75.0)	1 (25.0)	1.000^∥^
	Yes	224 (98.2)	147 (65.6)	77 (34.4)	
	TACE (± SR ± RFA ± PEIT)	180 (78.9)	119 (66.1)	61 (33.9)	
	TACE (± SR ± RFA) + sorafenib	24 (10.5)	13 (54.2)	11 (45.8)	
	TACE + chemotherapy^*^	9 (3.9)	4 (44.4)	5 (55.6)	
	SR	74 (32.5)	45 (60.8)	29 (39.2)	
	SR + sorafenib	11 (4.8)	5 (45.5)	6 (54.5)	
	Chemotherapy^†^	15 (6.6)	7 (46.7)	8 (53.3)	
	RFA	60 (26.3)	40 (66.7)	20 (33.3)	
Concurrent sorafenib	No	210 (92.1)	142 (67.6)	68 (32.4)	0.047^∥^
	Yes	18 (7.9)	8 (44.4)	10 (55.6)	
Post-RT treatment	No	64 (28.1)	43 (67.2)	21 (32.8)	0.781^∥^
	Yes	164 (71.9)	107 (65.2)	57 (34.8)	
	Sorafenib ± TACE ± RFA	77 (33.8)	50 (64.9)	27 (35.1)	
	TACE ± chemotherapy^‡^	89 (39.0)	56 (62.9)	33 (37.1)	
	Chemotherapy^§^	13 (5.7)	9 (69.2)	4 (30.8)	

Abbreviations: ECOG PS, Eastern Cooperative Oncology Group performance status; LC, liver cirrhosis; HBV, hepatitis B virus; AFP, alpha-fetoprotein; LN, lymph node; TACE, transarterial chemoembolization; SR, surgical resection; RFA, radiofrequency ablation; PEIT, percutaneous ethanol injection treatment; *RT*, radiotherapy

^*^5-Flurouracil plus Cisplatin (CDDP) (n = 3), Capecitabine (X) plus CDDP (n = 2), X plus Carboplatin (n = 1), X (n = 1), 5-Flurouracil plus Mitomycin C (n = 1), and Doxorubicin plus CDDP + X plus CDDP (n = 1)

^†^5-Flurouracil plus CDDP (n = 5), X plus CDDP (n = 4), X plus Carboplatin (n = 1), X (n = 1), 5-Flurouracil plus Mitomycin C (n = 1), and Doxorubicin plus CDDP + X plus CDDP (n = 1), Gemcitabine plus CDDP (n = 1), and Doxorubicin plus CDDP + 5-Flurouracil plus doxorubicin plus Mitomycin C (n = 1)

^‡^X plus CDDP (n = 2), 5-Flurouracil plus Leucovorin (n = 1), and Doxorubicin plus CDDP (n = 1)

^§^X plus CDDP (n = 6), 5-Flurouracil plus CDDP (n = 3), Gemcitabine plus CDDP (n = 1), 5-Flurouracil plus Leucovorin (n = 1), Doxorubicin plus CDDP (n = 1), and Doxorubicin (n = 1)

^∥^Fisher’s exact test.

^¶^t-test

### Treatment response and OS

After RT for metastatic abdominal LNs, LN responses were CR in 30 patients (13.2%), PR in 120 patients (52.6%), SD in 61 patients (26.8%), and PD in 17 patients (7.5%), yielding an ORR of 65.8%. ORR was similar in patients undergoing 3DCRT and IMRT/SBRT (63.7% [107/168] vs. 71.7% [43/60], *p*=0.264), and increased as radiation dose increased from ≤40 to 41 - 50 to >50 Gy_10_ (58.3% vs. 62.9% vs. 76.1%, *p*=0.077; Supplementary Figure 1). The patients with 1 to 2 metastatic LN(s) had a higher trend in median total radiation dose than the patients with ≥3 LNs (47.8 Gy_10_ [range, 31.3 - 80.0 Gy_10_] vs. 46.0 Gy_10_ [range, 30.0 - 65.0 Gy_10_], *p*=0.072) and also the patients with 1 to 2 metastatic LN had a higher trend in ORR than the patients with ≥3 metastatic LNs (73.8% or 66.7% vs. 59.4%, *p*=0.124). The distributions of various clinical parameters according to LN response are summarized in Table 1. ORR was significantly higher in patients with absence of vascular invasion and not receiving concurrent sorafenib than those of patients with presence of vascular invasion and receiving concurrent sorafenib (*p*<0.05 each), but the other clinical parameters were not significantly associated with LN response (*p*>0.05) (Table 1). The patients receiving concurrent sorafenib had greater frequencies of uncontrolled intrahepatic tumor (61.1% vs. 33.3%, *p*=0.022), distant metastasis (50% vs. 18.1%, *p*=0.004), and serum level of AFP of ≥ 400ng/mL (61.1% vs. 23.3%, *p*=0.001) than patients not receiving concurrent sorafenib (Supplementary Table 1).

**Figure 1:**
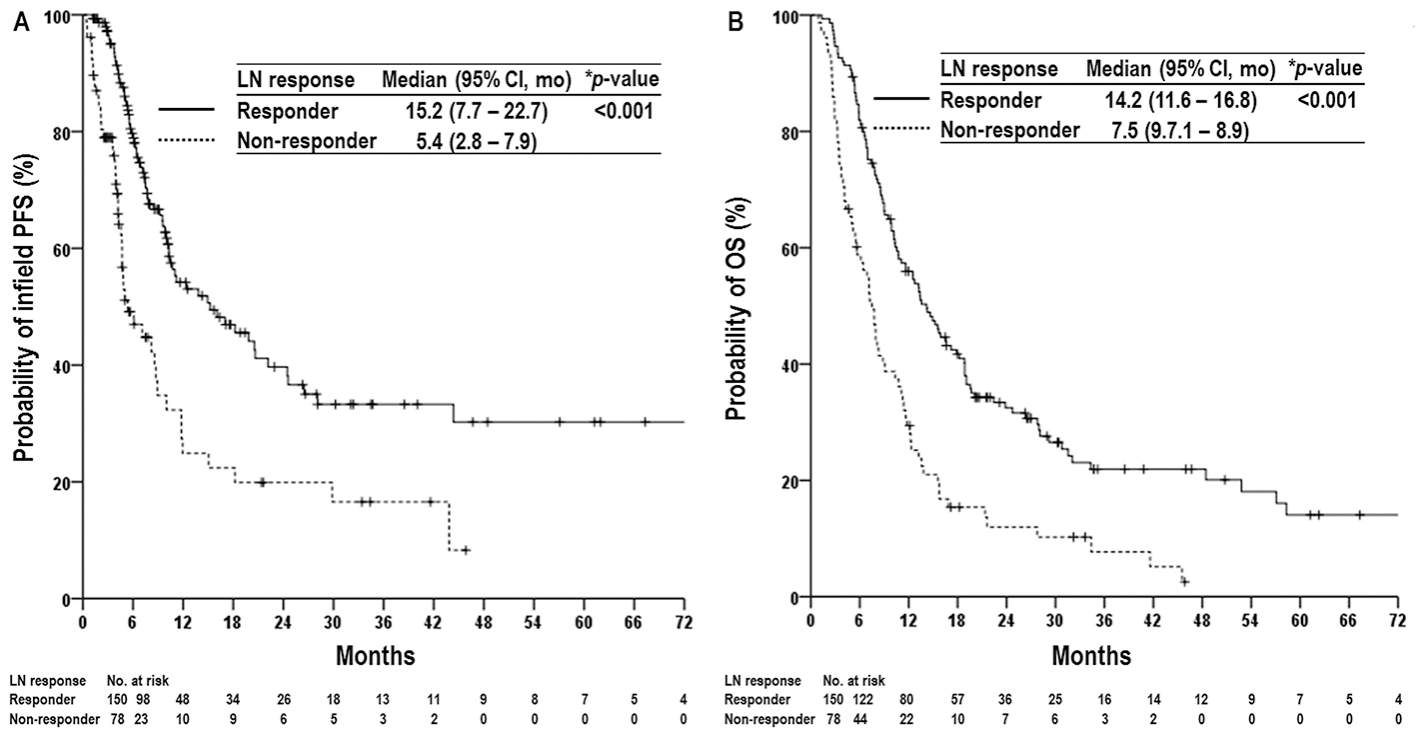
Infield progression-free survival (PFS) **(A)** and overall survival (OS) **(B)** curves in patients with and without a lymph node (LN) response. Abbreviations: Responder, complete or partial response; Non-responder, stable or progressive disease; CI, confidence interval; and mo, months. ^*^log-rank test.

At the time of analysis, 181 patients had died from disease, and 47 remained alive. The median follow-up duration for all patients was 10.6 months (range, 0.9 - 85.7 months). Of 228 patients, 210 (92.1%) developed disease progression, including 169 (74.1%) with intrahepatic progression, 147 (64.5%) with regional progression, and 138 (60.5%) with distant metastases. Of the 147 patients with regional progression, 118 had infield progression, and 83 had outfield progression, including 54 with both infield and outfield regional recurrence. The median times to infield and outfield progression were 5.7 months (range, 0.4 – 81.3 months) and 4.7 months (range, 0.8 – 41.4 months), respectively, and the median infield PFS and OS in all patients were 10.4 months (95% confidence interval [CI], 8.5 – 12.4 months) and 11.1 months (95% CI, 9.5 - 12.7 months), respectively. The actuarial 2-year infield PFS and OS rates were 33.6% (95% CI, 25.8 – 41.4%) and 25.5% (95% CI, 19.6 – 31.4%), respectively. Median OS times for patients receiving 3DCRT (n=168) and IMRT (n=59) were 10.3 months (95% CI, 8.6 – 12.0 months) and 14.6 months (95% CI, 11.1 – 18.1 months), respectively, and OS time of one patient receiving SBRT was 9.5 months (*p*=0.046). Of 150 patients who had ORR, 135 (90.0%) developed disease progression, including 107 (71.3%) with intrahepatic progression, 91 (60.7%) with regional progression, and 93 (62.0%) with distant metastases. Of the 91 patients with regional progression, 71 had infield progression, and 56 had outfield progression, including 36 with both infield and outfield regional recurrence. Of 78 patients who had no ORR, 75 (96.2%) developed disease progression, including 62 (79.5%) with intrahepatic progression, 56 (71.8%) with regional progression, and 45 (57.7%) with distant metastases. Of the 56 patients with regional progression, 47 had infield progression, and 27 had outfield progression, including 18 with both infield and outfield regional recurrence. LN responders showed significantly lower infield regional progression (47.3% [71/150] vs. 60.3% [47/78], *p*=0.026) and higher median infield PFS (15.2 months vs. 5.4 months, *p*<0.001) and OS (14.2 months vs. 7.5 months, *p*<0.001) than non-responders (Table 2) (Figure 1).

**Table 2:** Univariate and multivariate analysis of prognostic factor associated with overall survival (OS)

Factor		Univariate		Multivariate		
		OS, median (95% CI), months	*p* value^*^	HR	(95% CI)	*p* value^†^
Gender	Male	11.3 (9.1-13.4)	0.809	-	-	NS
	Female	9.9 (5.9-13.9)		-	-	
Age (years)	< 60	10.3 (8.3-12.4)	0.732	-	-	NS
	≥ 60	12.2 (9.8-14.7)				
ECOG PS	0-1	11.4 (9.7-13.0)	0.116	-	-	NS
	2-3	5.5 (2.2-8.8)				
Etiology of LC	HBV	9.1 (7.2-10.9)	0.012	-	-	NS
	Others	14.6 (12.0-17.3)		-	-	
Child-Pugh classification	A	12.3 (10.4-14.2)	< 0.001	1.000	-	
	B	5.5 (3.0-8.0)		2.590	(1.707-3.929)	< 0.001
AFP (ng/mL)	< 400	13.2 (11.2-15.2)	< 0.001	1.000	-	
	≥ 400	6.8 (5.3-8.3)		1.726	(1.208-2.467)	0.003
Vascular invasion	No	12.2 (9.1-15.3)	0.010	-	-	
	Yes	9.9 (7.9-11.9)		-	-	NS
Status of intrahepatic tumor	Controlled	14.6 (12.6-16.7)	< 0.001	1.000	-	
	Uncontrolled	6.4 (4.9-7.9)		3.037	(2.170-4.251)	< 0.001
Synchronicity of metastatic LN	No	13.2 (10.9-15.6)	< 0.001	-	-	
	Yes	6.2 (4.4-7.9)		-	-	NS
Size of metastatic LN (cm)	< 3	15.6 (11.5-19.6)	< 0.001	-	-	
	≥ 3	7.9 (6.4-9.3)		-	-	NS
No. of metastatic LN	1	15.6 (11.2-20.0)	< 0.001	1.000	-	
	2	12.5 (7.9-17.2)		1.220	(0.778 -1.914)	0.386
	≥ 3	7.8 (6.1-9.5)		1.832	(1.274-2.636)	0.001
Location of metastatic LN	Regional	13.3 (10.7-15.9)	< 0.001	1.000	-	
	Non-regional	7.1 (5.7-8.5)		1.889	(1.368-2.608)	< 0.001
Distant metastasis	Absent	11.8 (10.0-13.6)	< 0.001	1.000	-	
	Present	7.7 (4.2-11.2)		1.752	(1.223-2.509)	0.002
Previous treatment	No	5.5 (0.0-18.0)	0.303	-	-	
	Yes	11.1 (9.5-12.8)		-	-	NS
Concurrent sorafenib	No	11.6 (9.7-13.6)	0.058	-	-	NS
	Yes	5.2 (4.2-6.2)		-	-	
Post-RT treatment	No	8.5 (6.1-10.8)	0.723	-	-	NS
	Yes	11.6 (9.7-13.6)		-	-	
LN response	Responder	14.2 (11.6-16.8)	< 0.001	1.000	-	
	Non-responder	7.5 (6.1-8.9)		2.391	(1.741-3.282)	< 0.001

Abbreviations: CI, confidence interval; HR, Hazard Ratio; NS, not significant; Responder, complete or partial response; Non-responder, stable or progressive disease; all other abbreviations as in Table 1

^*^log-rank test

†Cox proportional hazards model.

**Figure 2:**
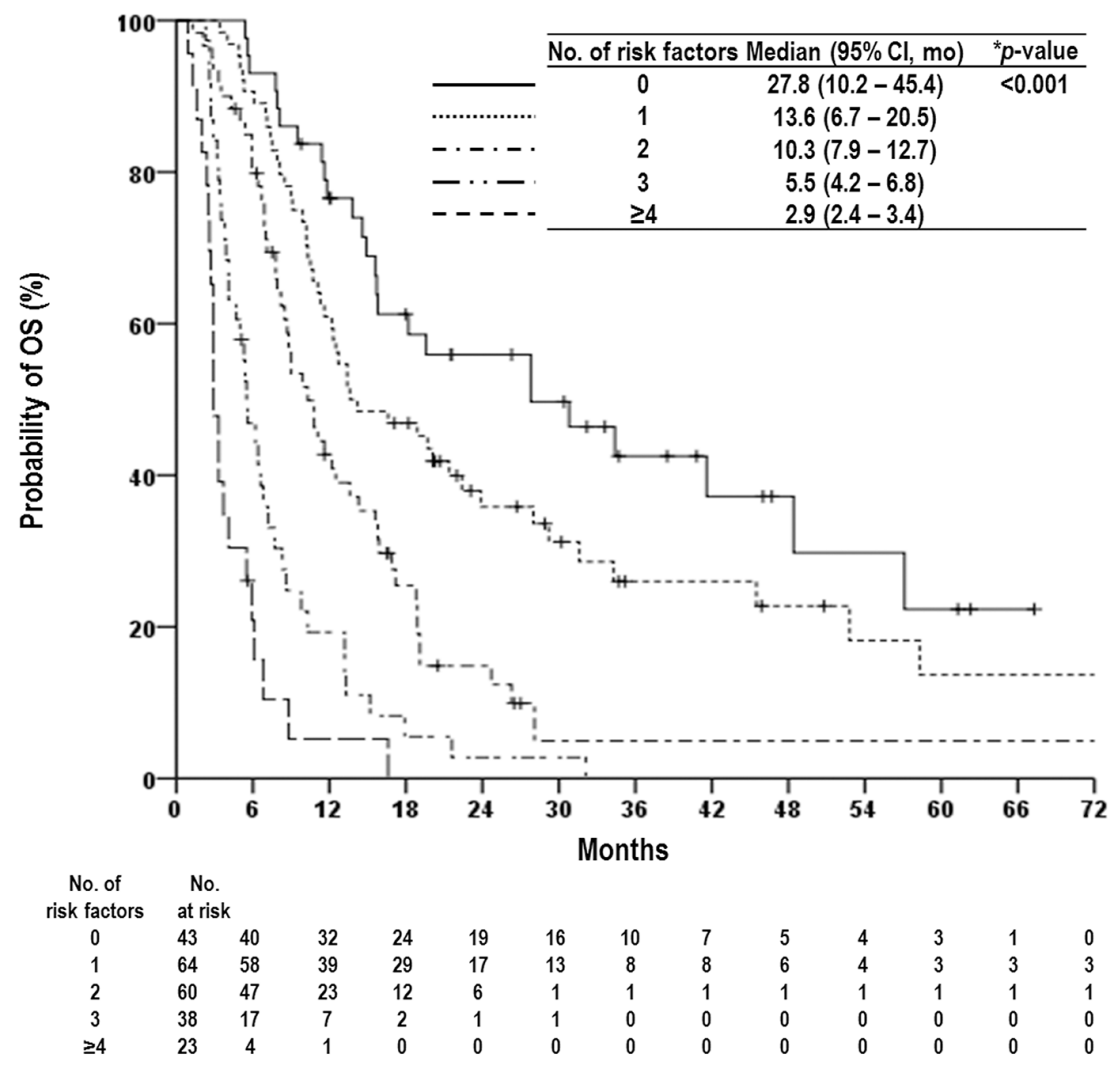
Overall survival (OS) curves according to prognostic group based on number of risk factors as follows: Child-Pugh classification, status of intrahepatic tumor, serum level of AFP, number of metastatic LNs, location of metastatic LNs, and the presence of distant metastasis Abbreviations: same as in Table 1 and 2. ^*^log-rank test.

Univariate and multivariate analyses were performed to identify parameters predicting OS (Table 2). Although univariate analyses showed that several factors were significantly associated with OS (*p*<0.05 each), multivariate analysis showed that Child-Pugh classification, serum level of AFP, status of intrahepatic tumor, number of metastatic LNs, location of metastatic LNs, presence of distant metastasis, and LN response were independently associated with OS (*p*<0.05 each). Given the results of the multivariate analysis, the patients were divided into five subgroups based on the number of pre-treatment risk factors: Child-Pugh classification, serum level of AFP, status of intrahepatic tumor, number of metastatic LNs, location of metastatic LNs, and the presence of distant metastasis. As the number of risk factors increased, OS significantly decreased (*p*<0.05) (Figure 2).

### Nomogram

**Figure 3:**
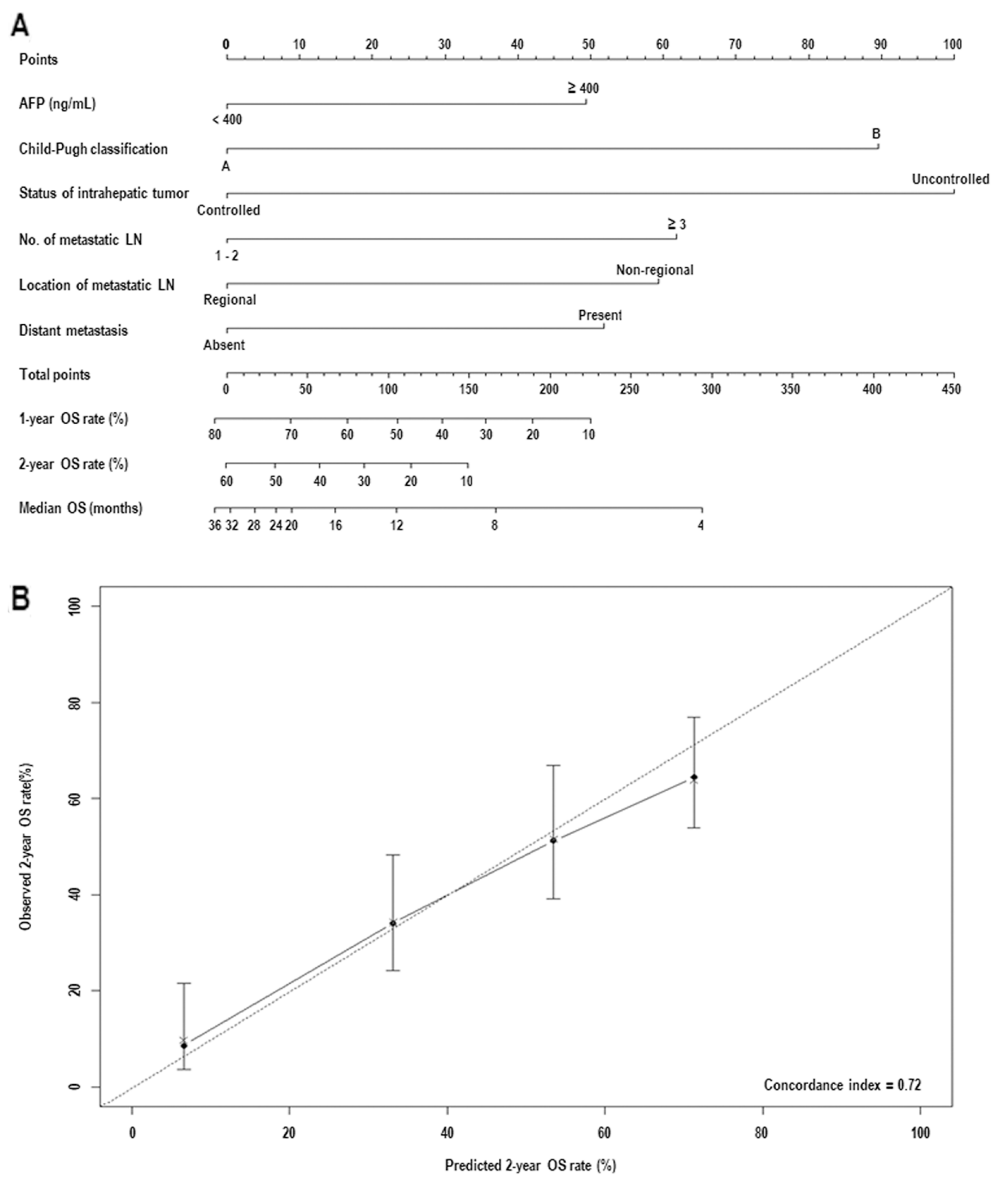
**(A)** Nomogram for the overall survival (OS) in hepatocellular carcinoma patients with abdominal lymph node (LN) metastasis. **(B)** Internal validation of accuracy for 2-year OS rate prediction based on the bootstrap validation method. Abbreviations: same as in Table 1 and 2.

Six pre-treatment risk factors associated with poor OS were used to develop a clinical nomogram (Figure 3A). The model was internally validated using the bootstrap validation method and demonstrated good accuracy in estimating the actual OS rate with a bootstrap-corrected C-index of 0.72 (Figure 3B). In addition, the values of C-index for each institution were similar: 0.68 for A and B institutions (n=78), 0.71 for C (n=81), and 0.76 for D (n=79).

### Toxicity

Of 228 patients, late gastrointestinal toxicities (GITs), defined as gastric or duodenal ulcers within the RT field, were observed in 27 patients (11.8%): grade 1 in 3 patients (1.3%), grade 2 in 11 patients (4.8%), and grade 3 in 13 patients (5.7%). The incidence of ≥2 grade GITs had no increasing trend with increasing radiation dose from ≤40 to 41-50 to >50 Gy_10_ (11.7% [7/60] vs. 10.3% [10/97] vs. 9.9% [7/71], *p*=0.941). No treatment-related hepatic failure or treatment-related death was observed.

## DISCUSSION

Despite recent advances in diagnostic techniques and therapeutic procedures, the prognosis for HCC patients with abdominal LN metastasis remains discouraging, with a median survival of less than 4 months without treatment [8, 10]. Based on the results of two randomized trials [8, 9], the HCC patients with abdominal LN metastasis, considered as an advanced staged disease, might be considered for sorafenib monotherapy rather than other local treatments. However, the absence of survival benefit in subgroups with extrahepatic metastases and low response rates (<5%) in patients receiving sorafenib monotherapy indicated that addition of less invasive and more effective local treatments would be needed in HCC patients with abdominal LN metastasis. In addition, regardless of the status of intrahepatic tumor and/or distant metastasis, local treatments to metastatic abdominal LNs could reduce the size or number of these LNs and subsequently could delay biliary, vascular, and bowel obstruction and facilitate subsequent treatments [3, 11, 13]. Thus, a higher objective response might be a prerequisite for the treatment of advanced HCC patients with metastatic abdominal LNs. Recent increases in the use of RT for HCC [12, 20–25] have led to the use of RT to treat metastatic abdominal LNs. Several studies of RT for HCC patients with LN metastasis showed promising outcomes, with ORRs of 66 to 100% for metastatic LNs and median OS times of 7 to 13 months [10, 12–19], and LN response to RT was found to be significantly associated with OS [12, 13, 17, 19]. Moreover, Chen et al. [18] reported that the patients who received RT had significantly longer median OS (9.4 months vs. 3.3 months, *p*<0.05) and lower incidence of LNs associated with death (8% vs. 43.5%, *p*<0.05) compared with those who did not receive RT. Similarly, we observed an ORR of 65.8% and a median OS of 11.1 months, with LN response to RT being a significant prognostic factor for infield PFS and OS. These results imply that RT of metastatic LN may help achieve tumor response and subsequently improve OS in advanced HCC patients with metastatic abdominal LNs.

It has been reported that baseline hepatic functions, such as Child-Pugh classification [10–12, 15, 16, 18, 19], the status of intrahepatic tumor and the presence of distant metastasis [12, 13, 15], and LN-related factors, such as location and number of metastatic abdominal LNs [7, 10, 18], were associated with OS in HCC patients with abdominal LN metastasis. In addition, based on retrospective evaluation of 435 Italian HCC patients, the Cancer of Liver Italian Program Group reported that serum level of AFP, most commonly used biomarker to assist in HCC diagnosis and to predict treatment response and prognosis, was one of the most important prognostic factors for HCC patients [26]. However, due that the number of study population in aforementioned studies was relatively small and single institutional, the reported prognostic factors for OS varied among studies [10–12, 15, 16, 18, 19]. Thus, we performed this retrospective multi-institutional study and showed that Child-Pugh classification, status of intrahepatic tumor, presence of distant metastasis, location and number of metastatic abdominal LNs, serum level of AFP, and the LN response to RT were significant prognostic factors on multivariate analysis. Prognostic group stratification according to number of pretreatment risk factors enables the clinician to easily stratify the patients depending on individual risk of death. In the present study, the median OS in patients with ≥ 4, 3, 2, 1, and 0 risk factors were 2.9, 5.5, 10.3, 13.6, and 27.8 months, respectively (*p*<0.05) (Figure 3). However, each risk factor has unequal impact on OS, i.e., hazard ratio for status of intrahepatic tumor, Child-Pugh classification, number of metastatic LNs, location of metastatic LNs, presence of distant metastasis, and serum level of AFP were 3.037, 2.590, 1.832, 1.889, 1.752, and 1.726, respectively (Table 2). Thus, a nomogram integrating the different prognostic contribution of each factor could be a more reliable tool to predict the OS of individual patients than prognostic group stratification according to number of risk factors. In the present study, C-index, widely used to evaluate the predictive accuracy, of a prognostic nomogram was 0.72, which was comparable or higher than those of traditional staging systems, such as Barcelona Clinic Liver Cancer, Cancer of the Liver Italian Program, and AJCC staging system [26, 27].

HCC patients with abdominal LN metastasis have a heterogeneous prognosis, depending on baseline liver function, the status of intrahepatic tumor, presence of distant metastasis, LN related factors, etc., and thus various treatments modalities have been attempted. Xiaohong et al. [7] showed that patients with a single metastatic LN had a significantly better OS than those with multiple metastatic LNs after surgical resection, and the median OS of patients with single metastatic LN was 29 months after surgical resection. Similarly, our findings showed that the median OS of patient with 1 to 2 metastatic LN(s), controlled intrahepatic tumor and Child-Pugh A was approximately 27.8 months after RT, with more favorable outcomes compared with the results (6.5 – 10.9 months) of sorafenib monotherapy in advanced HCC patients [8, 9]. Similar to other studies [10–13], we found that LN response to RT was significantly associated with OS, and ORR increased with increase in radiation dose, being 58.3%, 62.9%, and 76.1% in patients receiving radiation doses of ≤40 to 41-50 to >50 Gy_10_, respectively, with low incidence (5.7%) of grade ≥3 GIT. In addition, although ORR was similar in patients undergoing 3DCRT and IMRT, the patients receiving IMRT had significant higher OS than those receiving 3DCRT. These findings suggested that higher radiation doses using modern RT techniques, such as IMRT, might be more effective in achieving tumor control in patients with fewer risk factors. Fortunately, multikinase inhibitors, such as sorafenib, have been found to prolong survival in patients with advanced HCC [8, 9] and may enhance tumor sensitivity to RT [28]. Although the present study did not show the clinical benefits of multikinase inhibitors due to the high frequencies of unfavourable pre-treatment factors, such as uncontrolled intrahepatic tumor, distant metastasis, and AFP of ≥ 400ng/Ml, in patients receiving sorafenib, aforementioned studies suggest that sequential or concurrent use of multikinase inhibitors with RT may prevent or delay intrahepatic or extrahepatic spread as well as enhance the tumor sensitivity to RT to metastatic LNs. Larger scaled prospective randomized studies comparing multikinase inhibitors with RT plus multikinase inhibitors are warranted in these patients. However, because our data were from retrospective study and did not include the patients who did not receive RT, the effects of local and systemic treatment for intrahepatic and/or metastatic disease and probable selection bias were not thoroughly evaluated. In addition, our data was not externally validated using the validation cohort. Nevertheless, the present study has several strengths. First, the entire study cohort completed RT. Second, tumor response was evaluated by imaging studies. Third, to the best of our knowledge, a relatively large sample size was achieved through the use of a multi-institutional study and high accuracy of our formulated prognostic models make this study worthwhile for predicting OS in HCC patients with abdominal LN metastasis and choosing treatment strategies for these patients.

In conclusion, our data showed that Child-Pugh classification, the status of intrahepatic tumor, number of metastatic LN, location of metastatic LN, presence of distant metastasis, serum level of AFP, and LN response to RT were significant prognostic factors associated with OS in HCC patients with abdominal LN metastasis and suggested that the prognostic group stratification according to number of pre-treatment risk factors and nomogram formulated in present study could be useful prognostic indicators predicting the OS and therapeutic indicators when choosing treatment strategies for these patients, such as supportive care, surgical resection, RT, sorafenib, and aggressive multimodal approaches.

## MATERIALS AND METHODS

This retrospective study was performed using the medical records of 230 patients receiving RT for abdominal LN metastases from HCC between June 2008 and December 2013 in four institutions of the KROG. The inclusion criteria were as follows: (i) HCC was diagnosed based on the guidelines of the Korean Liver Cancer Study Group and the National Cancer Center [12, 29] ([1] histological confirmation; [2] the presence of risk factors, including hepatitis B virus [HBV], hepatitis C virus [HCV] or liver cirrhosis [LC], serum ɑ-fetoprotein [AFP] concentration ≥ 200 IU/mL, and HCC-compatible radiological features on one or more imaging modalities, such as computed tomography [CT], magnetic resonance imaging [MRI], and/or angiography; or [3] the presence of risk factors, including HBV, HCV, or LC; serum AFP < 200 IU/mL, and HCC-compatible radiological features on two or more imaging modalities); (ii) The abdominal metastatic LNs were pathologically proven (n=12) or clinically detected by the following radiological findings (n=218): ([1] short-axis diameter of contrast-enhanced LN ≥ 1 cm on CT and/or MRI; and [2] increase in size over time); (iii) completion of planned RT; (iv) RT with modern techniques, including three-dimensional conformal radiotherapy (3DCRT), intensity-modulated RT (IMRT), or stereotactic body RT (SBRT); (v) no history of malignancy other than HCC within 5 years; (vi) no history of previous irradiation to abdominal LNs. Of the 230 patients who met the criteria, 2 who had no medical record immediately after RT were excluded. The remaining 228 patients were analyzed in this study. Decision of RT and combinations and sequence with other treatments was made by each institutional policy considering the status of intrahepatic tumor, presence of distant metastasis, baseline liver function, etc. The present study was approved by the KROG and the institutional review board of each participating institution, and written informed consent was waived due to the retrospective nature of the study.

Data regarding patients, tumor, treatment, survival, and treatment failure were collected. Treatment data included RT technique, total radiation dose, fractional dose, and other treatment modalities. Total radiation doses were converted to equivalent doses in 2 Gy fraction (EQD2), calculated using a linear quadratic model with a ɑ/β ratio of 10. The synchronicity of metastatic LN was defined as less than 1 month of a diagnostic interval between intrahepatic primary tumor and LN metastasis. Abdominal LNs were divided into regional and non-regional LNs based on the American Joint Committee on Cancer (AJCC) guidelines. Regional LNs were defined as hilar, hepatoduodenal ligament, inferior phrenic and caval LNs around the hepatic artery, portal vein and celiac trunk, while abdominal LNs outside the regional LN area were considered as non-regional. The responses of the metastatic LNs were defined as the maximal tumor response observed during the follow-up period using the Response Evaluation Criteria in Solid Tumors (RECIST version 1.1) [30] using CT and/or MRI. A complete response (CR) was defined as the disappearance of the all target lesions, a partial response (PR) was defined as a ≥ 30% reduction in the sum of diameters of target lesions, progressive disease (PD) was defined as a ≥ 20% increase in the sum of diameters of target lesions or the appearance of one or more new lesions in RT fields, and stable disease (SD) was defined as a response that did not qualify as a PR or PD. The objective response rate (ORR) was defined as the sum of the CR and PR rates. Treatment-related toxicities were scored using Common Terminology Criteria for Adverse Events version 3.0. Disease progression was determined pathologically by biopsy or cytology and/or radiological findings, showing an increase in size over time. Regional progression was defined as a regrowth or new tumor in abdominal nodal area, intrahepatic progression was defined as a regrowth or new intrahepatic tumor, and distant metastasis was defined as peritoneal seeding or metastasis to extra-abdominal sites. Regional progression was divided into infield and outfield regional progression: infield regional progression was defined as a regrowth or new tumor within the treated volume, and outfield regional progression was defined as a regrowth or new tumor in the abdominal nodal area outside of the treated volume. Infield progression-free survival (PFS) and overall survival (OS) were defined as the intervals from the date of the start of RT to the date of detection of infield progression and death or last follow-up, respectively. OS rates were calculated using the Kaplan-Meier method. Univariate analysis of parameters predicting OS were assessed with log rank tests, followed by multivariate analysis using Cox’s proportional hazard model with a stepwise forward procedure. Statistical analyses were two-sided and were performed using STATA software (version 9.0; Stata Corp., College Station, TX). A *p* value <0.05 indicated statistical significance. The nomogram was formulated by R (version 3.3.2, http://www.r-project.org) according to the results of the multivariate analysis. The predictive accuracy of the nomogram for OS was measured by concordance index (C-index), and the calibration was generated by 1000 bootstrap samples to decrease the bias. For clinical use of the nomogram, after the points at which each variable was counted, the total points were calculated for assessing the probability of the survival rate.

## SUPPLEMENTARY MATERIALS FIGURE AND TABLE


