# Supplementary material for: Prognostic group stratification and nomogram for predicting overall survival in patients who received radiotherapy for abdominal lymph node metastasis from hepatocellular carcinoma: a multi-institutional retrospective study (KROG 15-02)
Source: Oncotarget. 2017 Oct 10;8(55):94450–61. doi: 10.18632/oncotarget.21775 (PMC5706887; doi:10.18632/oncotarget.21775)
Supplement: Supplementary file 1 [file oncotarget-08-94450-s001.pdf]

Prognostic group stratification and nomogram for predicting overall survival in patients who received radiotherapy for abdominal lymph node metastasis from hepatocellular carcinoma: a multi-institutional retrospective study (KROG 15-02)

SUPPLEMENTARY MATERIALS

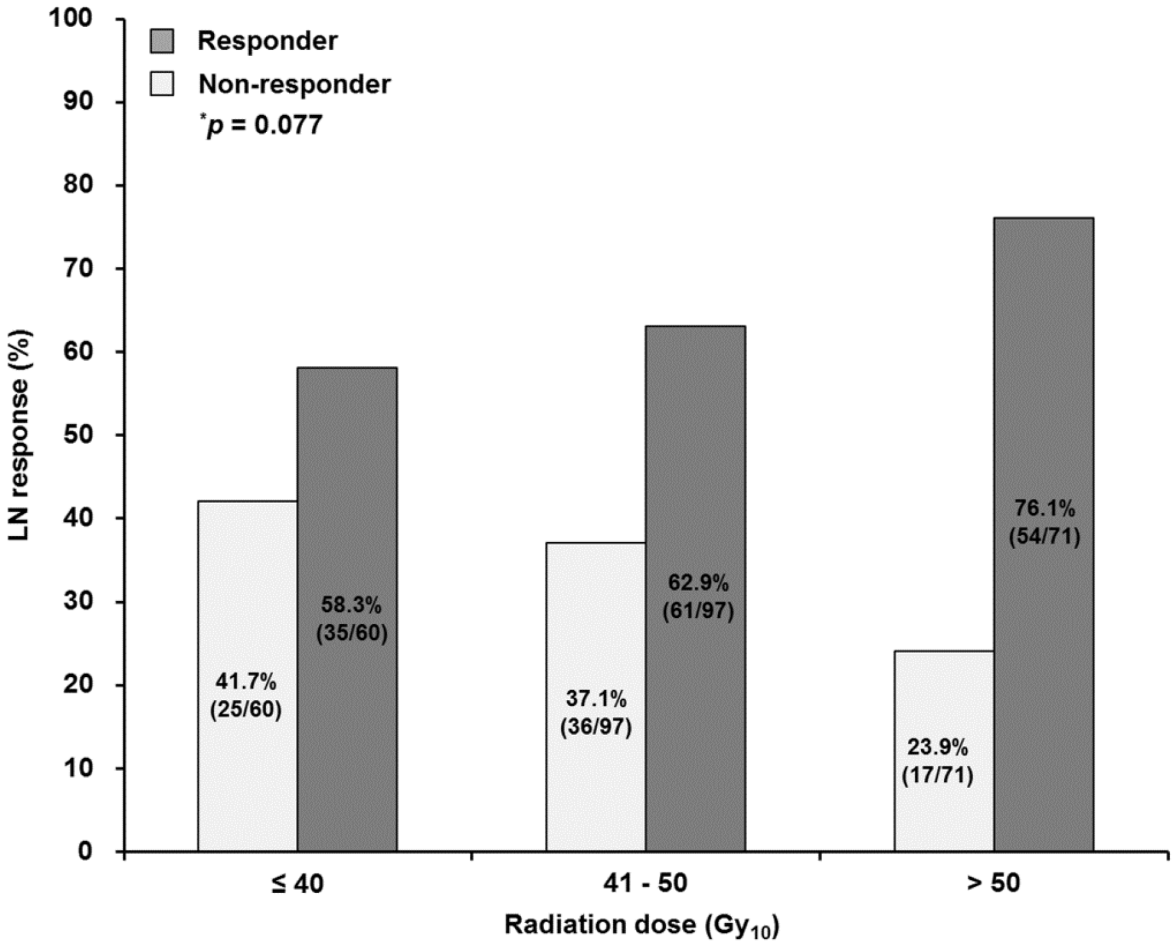

Supplementary Figure 1: Relationship between lymph node (LN) response and radiation dose. Abbreviations: Responder, complete or partial response; and Non-responder, stable or progressive disease. *Fisher's exact test.

Supplementary Table 1: Distribution of pre-treatment factors between two groups whether received concurrent sorafenib or not

Characteristics		Concurrent sorafenib, n (%)		*p* value*
		No	Yes	
Gender	Male	178 (84.8)	16 (88.9)	0.749
	Female	32 (15.2)	2 (11.1)	
Age (years)	< 60	105 (50.0)	13 (72.2)	0.087
	≥ 60	105 (50.0)	5 (27.8)	
ECOG PS	0-1	200 (95.2)	17 (94.4)	1.000
	2-3	10 (4.8)	1 (5.6)	
Etiology of LC	HBV	139 (66.2)	13 (72.2)	0.796
	Others	71 (33.8)	5 (27.8)	
Child-Pugh classification	A	182 (86.7)	14 (77.8)	0.481
	B	28 (13.3).	4 (22.2)	
AFP (ng/mL)	<400	161 (76.7)	7 (38.9)	0.001
	≥400	49 (23.3)	11 (61.1)	
Vascular invasion	No	159 (75.7)	10 (55.6)	0.089
	Yes	51 (24.3)	8 (44.4)	
Status of Intrahepatic tumor	Controlled	140 (66.7)	7 (38.9)	0.022
	Uncontrolled	70 (33.3)	11 (61.1)	
Synchronicity of metastatic LN	No	163 (77.6)	13 (72.2)	0.770
	Yes	47 (22.4)	5 (27.8)	
Size of metastatic LN (cm)	≤ 3	110 (52.4)	5 (27.8)	0.052
	> 3	100 (47.6)	13 (72.2)	
No. of metastatic LN	1	74 (35.2)	6 (33.3)	1.000
	2	39 (18.6)	3 (16.7)	
	≥ 3	97 (46.2)	9 (50.0)	
Location of metastatic LN	Regional	143 (68.1)	10 (55.6)	0.302
	Non-regional	67 (31.9)	8 (44.4)	
Distant metastasis	Absent	172 (81.9)	9 (50.0)	0.004
	Present	38 (18.1)	9 (50.0)	

Abbreviations: as in Table 1
*Fisher's exact test.
